# Artificially Selected Grain Shape Gene Combinations in Guangdong Simiao Varieties of Rice (*Oryza sativa* L.)

**DOI:** 10.1186/s12284-023-00620-9

**Published:** 2023-01-17

**Authors:** Tifeng Yang, Haiyong Gu, Wu Yang, Bin Liu, Shihu Liang, Junliang Zhao

**Affiliations:** 1 Rice Research Institute, Guangdong Academy of Agricultural Sciences, Guangzhou, 510640 China; 2 Guangdong Key Laboratory of New Technology in Rice Breeding, Guangzhou, 510640 China; 3 Guangdong Rice Engineering Laboratory, Guangzhou, 510640, China

**Keywords:** Guangdong Simiao varieties, Grain shape, Fixation index, Allele mining, Molecular breeding, Rice

## Abstract

**Background:**

Grain shape is a key trait in rice breeding. Although many QTLs and genes of grain shape have been identified, how different combinations of alleles of these genes affect grain shape is largely unknown. It is important to understand the effects of grain shape gene combinations for breeding by design. In the present study, we performed genetic dissection of the grain shapes in Guangdong Simiao varieties, a popular kind of rice in South China, to identify the effective alleles and their combination for breeding.

**Results:**

We selected two hundred nineteen *indica* accessions with diverse grain shapes and fifty-two Guangdong Simiao varieties with long and slender grain shapes for genome-wide selection analysis. The results showed that four (*GS3*, *GS5*, *GW5 *and *GL7*) of the twenty grain shape genes fall into the regions selected for in Guangdong Simiao varieties. Allele analysis and frequency distribution of these four genes showed that *GS3*^allele3^ and *GW5*^allele2^ accounted for 96.2%, and *GL7*^allele2^ and *GS5*^allele2^ accounted for 76.9% and 74.5% of the Simiao varieties, respectively. Further analysis of the allelic combinations showed that 30 allelic combinations were identified in the whole panel, with 28 allelic combinations found in the international *indica* accessions and 6 allelic combinations found in Guangdong Simiao varieties. There were mainly three combinations (combinations 17, 18 and 19) in the Guangdong Simiao varieties, with combination 19 (*GS3*^allele3^ + *GW5*^allele2^ + *GL7*^allele2^ + *GS5*^allele2^) having the highest percentage (51.9%). All three combinations carried *GS3*^allele3^ + *GW5*^allele2^, while combinations 17 (*GL7*^allele1^) and 19 (*GL7*^allele2^) showed significant differences in both grain length and length/width ratio due to differences in *GL7* alleles. Pedigree analysis of Guang8B, the maintainer of the first released Simiao male sterile line Guang8A, showed that the parent lines and Guang8B carried *GS3*^allele3^ + *GW5*^allele2^ + *GS5*^allele2^, while the *GL7* allele differed, resulting in significant differences in grain size.

**Conclusion:**

The results suggest that specific alleles of *GS3*, *GS5*, *GW5* and *GL7* are the key grain shape genes used in the Guangdong Simiao varieties and selected for grain shape improvement. Combination 19 is the predominant allelic combination in the Guangdong Simiao varieties. Our current study is the first to dissect the genetics of grain shape in Guangdong Simiao varieties, and the results will facilitate molecular breeding of Guangdong Simiao varieties.

**Supplementary Information:**

The online version contains supplementary material available at 10.1186/s12284-023-00620-9.

## Background

Rice (*Oryza sativa* L.) is one of the most important food crops worldwide, feeding more than half of the world's population. Grain shape is a key determinant of grain yield, grain quality and market value (Xing and Zhang 2010; Zuo and Li 2014) and is characterized by a combination of grain length, grain width and length/width ratio. The processing quality, cooking quality and taste quality of rice are also closely related to grain shape (Huang et al. 2013). Due to its great importance in determining the yield and quality of rice, grain shape has long been a key target trait in rice breeding (Meyer and Purugganan 2013).

Consumer preferences for rice grain shape vary across regions. Most people from Southern China, India, Thailand, Vietnam, the Philippines, Malaysia, Indonesia, and Pakistan prefer slender grains, while people from northern China, Japan, Korea, and Sri Lanka prefer short-grained varieties (Bai et al. 2010; Harberd 2015). Generally, rice grains with comparatively long and slender shapes are highly prized in many parts of the world, since slender grains tend to be transparent and lack opaque patches that are associated with an unpleasant chalky texture and taste (Harberd 2015).

Grain shapes in rice are a series of complex quantitative traits, including grain length, grain width, and length/width ratio, which are controlled by multiple genes (Zuo and Li 2014). With the advancement of genomic and genetic technologies, great progress has been made in resolving the genetic basis of grain shape. At present, twenty genes have been reported to control grain shape in rice, including *GL1* (Zhang et al. 2021b)*, GW2* (Song et al. 2007)*, GS2* (Duan et al. 2015; Hu et al. 2015)*, GS3* (Fan et al. 2006; Zhang et al. 2021b)*, qGL3/GL3.1* (Qi et al. 2012; Zhang et al. 2012)*, TGW3/qTGW3/GL3.3* (Hu et al. 2018; Xia et al. 2018; Ying et al. 2018), *LGY3* (Liu et al. 2018), *GW5/GSE5/qSW5* (Shomura et al. 2008; Weng et al. 2008; Duan et al. 2017; Liu et al. 2017)*, GS5* (Li et al. 2011)*, GW5.1* (Zhang et al. 2021b)*, GS6* (Sun et al. 2013)*, TGW6* (Ishimaru et al. 2013)*, GW6a* (Song et al. 2015)*, GW6* (Shi et al. 2020; Tang et al. 2021), *GLW7* (Si et al. 2016)*, GW7/GL7* (Wang et al. 2015a, b)*, GW8* (Wang et al. 2012)*, GS9/GL9* (Zhao et al. 2018; Lin et al. 2022)*, GW10* (Zhan et al. 2021) and *GL10* (Zhan et al. 2022). However, although many QTLs and genes controlling grain shape in rice have been identified, how different combinations of the alleles of these genes affect grain shape and are selected during the breeding process to acquire desired grain shapes is largely unknown. Therefore, obtaining different allelic combinations of grain shape genes is essential for yield improvement, potentially enabling breeders to develop high-yielding varieties with specific morphological characteristics of grain to satisfy diverse quality requirements (Fitzgerald et al. 2009).

The Guangdong Simiao varieties are a series of high-quality *indica* varieties with many distinctive local characteristics, among which the long and slender grain shape is one of the most essential traits. According to the Guangdong Simiao Rice Alliance, the grain shape standard of brown rice for Guangdong Simiao rice is grain length > 6.5 mm and length/width ratio > 3.5 (Group standards of the Guangdong seed association 2019). The Guangdong Simiao varieties showed very high grain quality, at least partially due to their long and slender grain shape. The Guangdong Simiao variety has a long history and has been recorded in China since the Qing Dynasty for over 200 years. Guangdong Simiao rice is so famous for its high quality that there is a saying in China, "The Simiao rice in southern China is like the pearl and jewel of high-quality rice". Guangdong Simiao rice is also one of the most popular export commodities, exported to more than 20 countries and regions, including Singapore, Malaysia, Hong Kong and Macao, Western Europe, North America and Africa (Wang et al. 2021). In the past two decades, significant progress has been made in Guangdong Simiao rice breeding. Sixteen Guangdong Simiao varieties, such as Meixiangzhan 2, Xiangyaxiangzhan, and 19 Xiang, have been released in Guangdong, China, based on the standards of Guangdong Simiao rice. Great achievements have also been made in Guangdong Simiao hybrid rice breeding. Guang8A is the first male sterile line for Guangdong Simiao hybrid rice in China, and a series of high-quality Simiao hybrid varieties have been released by using Guang8A as the male sterile line, with a total planted area of 1.1 million hectares.

Given the impact of its achievements in breeding high quality and improved grain shape in rice, Guangdong Simiao varieties are valuable materials to explore grain shape gene combinations in the scope of genomics and genetics. It is obvious that the long and slender grain shape of Guangdong Simiao varieties has been achieved by intensive artificial selection during the breeding process (Zhou et al. 2022). However, the breeding process and allelic selection for the grain shape gene are currently largely unknown. Clarifying which grain shape-related genes were selected and whether specific allelic combinations of these genes were used to achieve the characteristic grain shape in the Guangdong Simiao varieties are of great practical significance for developing breeding programs to improve grain shape in rice.

Facilitated by next-generation sequencing technologies and genomic tools, the comparison of large-scale rice genomes has become a reality (Xie et al. 2015; Zhang et al. 2021a). This provides a series of genomic tools for unraveling the genetic base of complex traits in rice. In this study, we presented a landscape of allele selection and distribution of grain shape genes in Simiao varieties by comparing the genome-wide variations between Simiao varieties and an *indica* accession panel representing international diversity using whole genome deep resequencing data. Combined with the grain shape phenotype data, we successfully identified four genes (*GS3*, *GS5*, *GW5* and *GL7)* that were selected in Guangdong Simiao varieties. We also characterized a specific allelic combination of these four genes that was predominant in Guangdong Simiao varieties. These results were further confirmed using the pedigree of Guang8B, the maintainer line of the first released Guangdong Simiao-type male sterile line Guang8A. The present study provided a comprehensive study of the selection pattern of grain shape genes, as well as their allele combinations in Guangdong Simiao varieties for their grain shape and quality improvement.

## Results

### The Differences in Grain Shape Phenotypes Between the International Indica Accessions and Guangdong Simiao Varieties

**Fig. 1:**
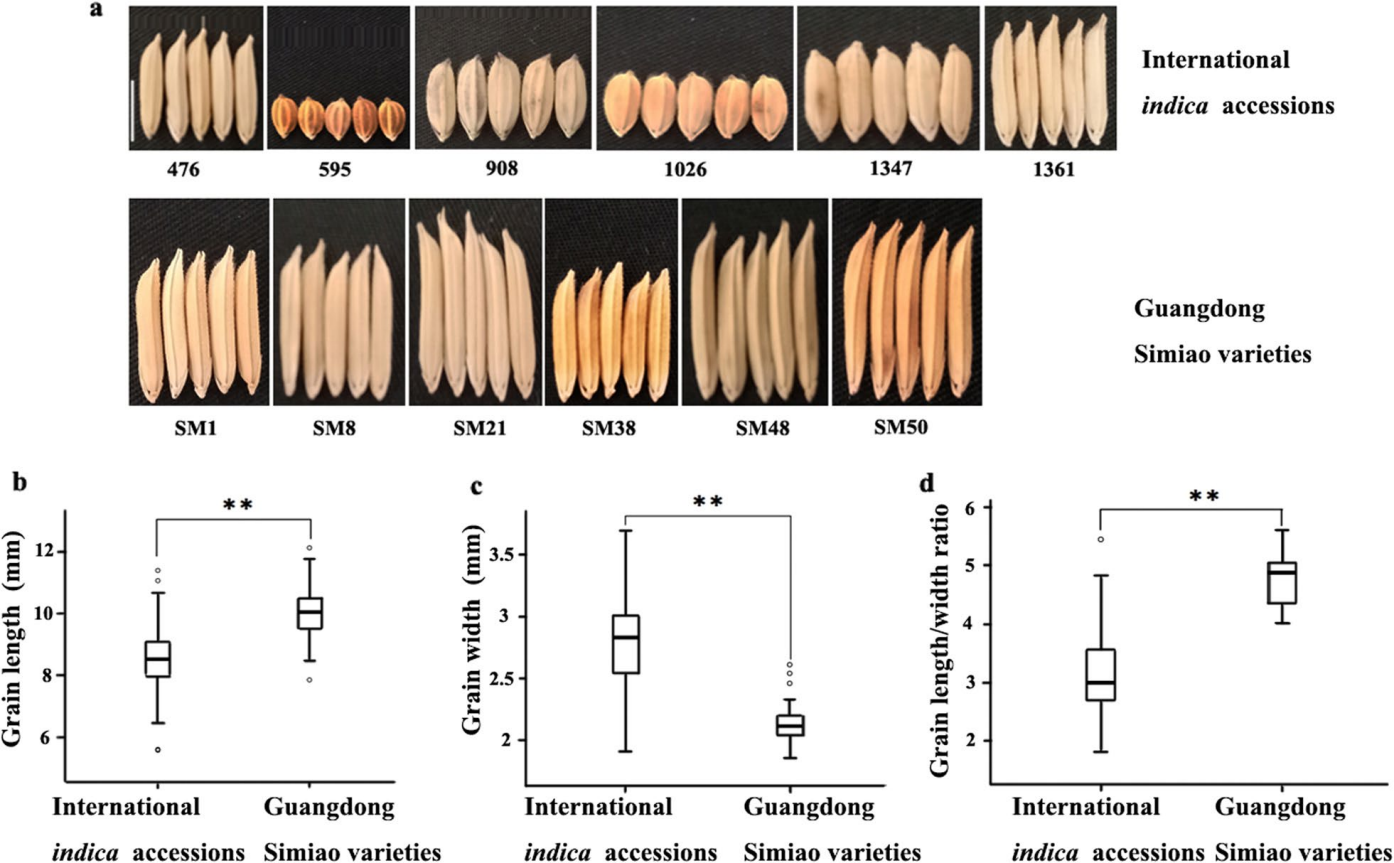
Grain shape differences between international *indica* accessions and Guangdong Simiao varieties. **a** There were great differences between international *indica* accessions and Guangdong Simiao varieties in grain shape; the differences in grain length (**b**), grain width (**c**) and length/width ratio (**d**) between international *indica* accessions and Guangdong Simiao varieties.Scale bar:5mm; ** *P* < 0.01; the black horizontal lines represent the median value. The upper side and lower side of the box represent the upper quartile and lower quartile, respectively. The whiskers represent the range of data, and small circles represent outliers

The grain shape phenotypes, including grain length, grain width and length/width ratio, of fifty-two Guangdong Simiao varieties and two hundred nineteen *indica* accessions from different countries were assessed in this study (Fig. 1). For the international *indica* accessions, the grain length ranged from 5.58 to 11.38 mm, with an average of 8.59 mm and a variation coefficient of 10.58%; the grain width ranged from 1.91 to 3.69 mm, with an average of 2.79 mm and a variation coefficient of 11.57%; and the length/width ratio ranged from 1.82 to 5.45, with an average of 3.14 and a variation coefficient of 18.86%. For the Guangdong Simiao varieties, the grain length ranged from 7.85 to 12.11 mm, with an average of 10.01 mm and a variation coefficient of 8.26%; the grain width ranged from 1.86 to 2.61 mm, with an average of 2.13 mm and a variation coefficient of 6.94%; and the length/width ratio ranged from 4.02 to 5.61, with an average of 4.77 and a variation coefficient of 8.90% (Fig. 1, Additional file 1: Table S1). Significant differences in grain length, grain width and length/width ratio were detected between the international *indica* accessions and Guangdong Simiao varieties. The grains of Guangdong Simiao varieties are more slender (lower grain width, higher grain length) than the international *indica* accessions, which is characteristic of Guangdong Simiao varieties (Fig. 1).

### Grain Shape Genes Selected in Guangdong Simiao Varieties

**Fig. 2:**
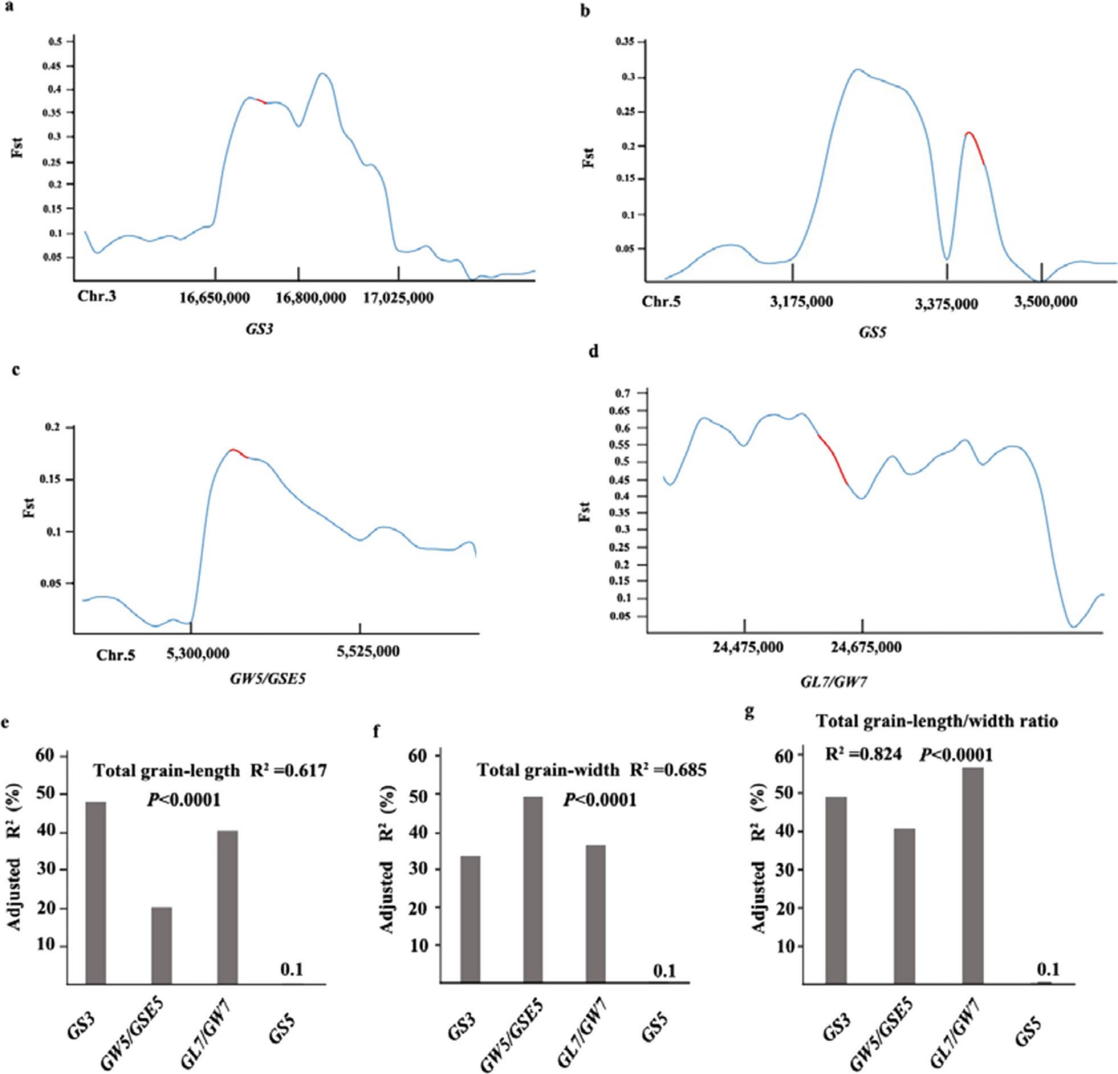
Fixation index of genomic regions harboring four grain shape genes and the phenotypic variation explanation of these four genes in the whole panel. The red line: the genome regions harboring the target grain shape gene; the Fst of *GS3* (**a**), *GS5* (**b**), *GW5/GSE5* (**c**) and *GL7/GW7* (**d**); the contribution of these four genes (*GS3*, *GW5/GSE5*, *GL7/GW7* and *GS5*) to grain length (**e**), grain width (**f**) and length/width ratio (**g**)

To identify the grain shape genes specifically selected by Guangdong Simiao varieties, fixation index (Fst) values were calculated between the international *indica* accessions and Guangdong Simiao varieties based on the whole genome resequencing data. The selection signals were identified according to Fst values. Further analysis was conducted to identify colocalization between previously characterized grain shape genes and the selection signals. The results showed that four (*GS3*, *GS5*, *GW5* and *GL7*) out of the twenty grain shape genes fall into the highest 10% Fst regions, with the highest Fst value of 0.52 in which *GL7* is located (Fig. 2a–d). The remaining sixteen genes were located in the genomic segments with Fst values lower than 0.15, suggesting that they might not be under intensive artificial selection during the breeding process of Guangdong Simiao varieties (Additional file 2: Table S2).

We further assessed the genetic contributions of the four genes to grain shape in the whole panel by regression analysis. The four grain shape genes could explain 61.7%, 68.5% and 82.4% of the phenotypic variations in grain length, grain width and length/width ratio, respectively (Fig. 2e–g). Among these genes, *GS3* accounted for 47.9% of the grain length variation; *GW5* accounted for 49.3% of the grain width variation; *GL7* accounted for 56.9% of the length/width ratio variation, respectively; and the other genes showed diverse effects on grain shapes (Fig. 2e–g).

### Allelic Effects of the Four Genes on Grain Shape Phenotypes of the Rice Accessions Tested

**Fig. 3:**
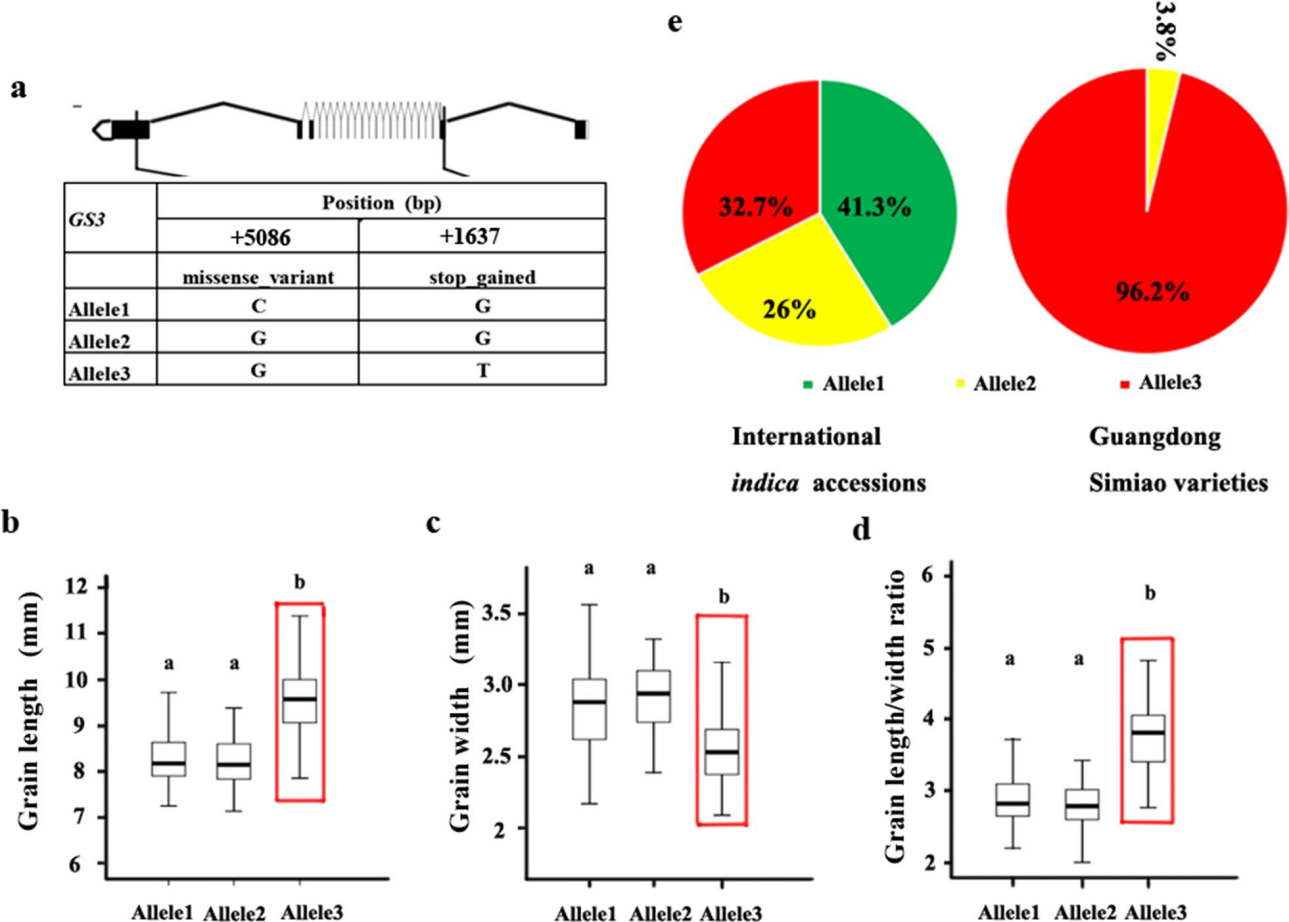
Allelic effects and distribution of *GS3*. Alleles of *GS3* (**a**); comparisons of grain length (**b**), grain width (**c**) and length/width ratio (**d**) for the three *GS3* alleles; the distribution of *GS3* alleles (**e**) in international *indica* accessions and Guangdong Simiao varieties; letters above the bars are ranked by Duncan’s test at *P* < 0.05; different letters indicate significant differences; red rectangle box: major allele in Guangdong Simiao varieties; scale bar: 100 bp

**Fig. 4:**
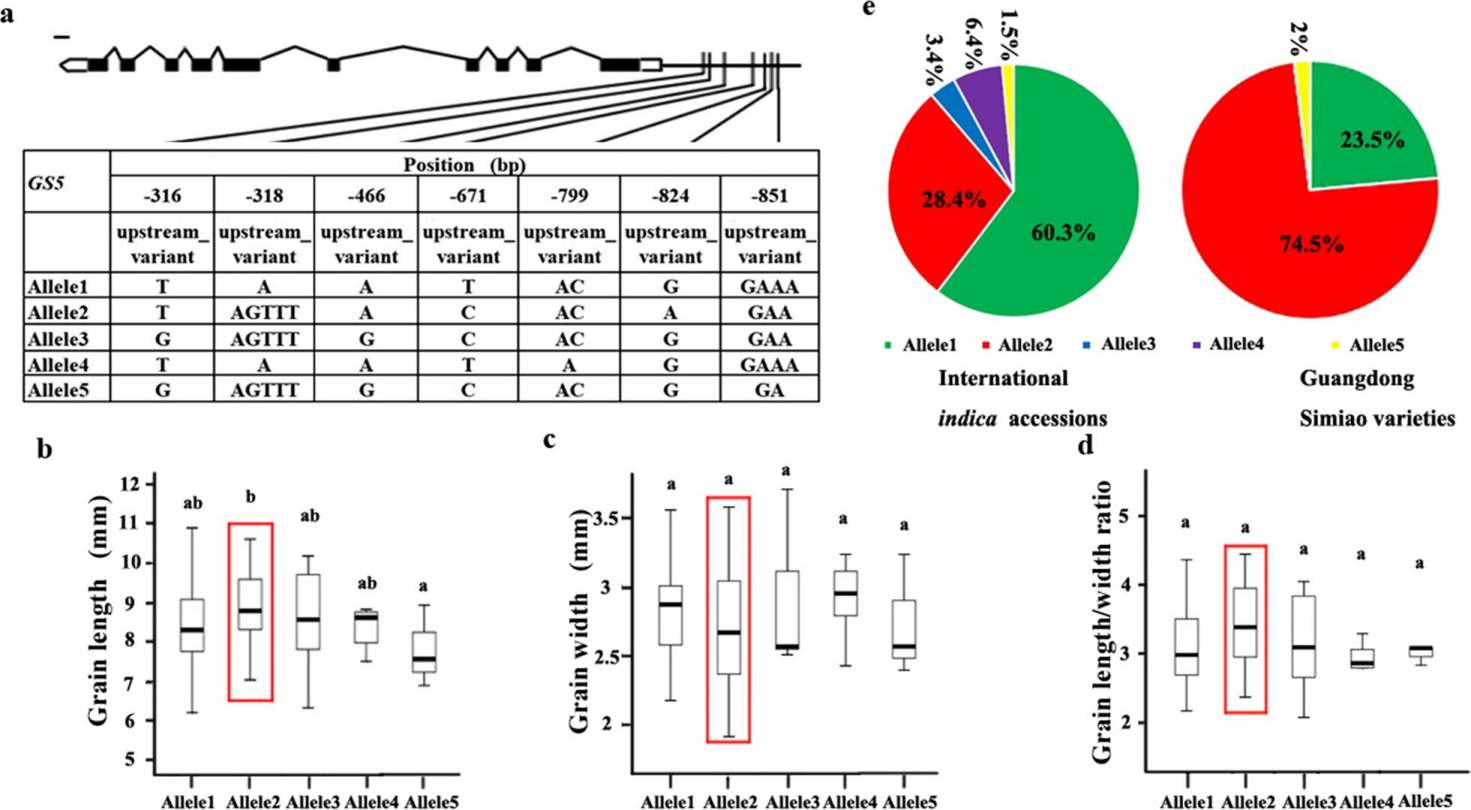
Allelic effects and distribution of *GS5*. Alleles of *GS5* (**a**); comparisons of grain length (**b**), grain width (**c**), and length/width ratio (**d**) for the five *GS5* alleles; the distribution of *GS5* alleles (**e**) in international *indica* accessions and Guangdong Simiao varieties; letters above the bars are ranked by Duncan’s test at *P* < 0.05; different letters indicate significant differences; red rectangle box: major allele in Guangdong Simiao varieties; scale bar: 100 bp

**Fig. 5:**
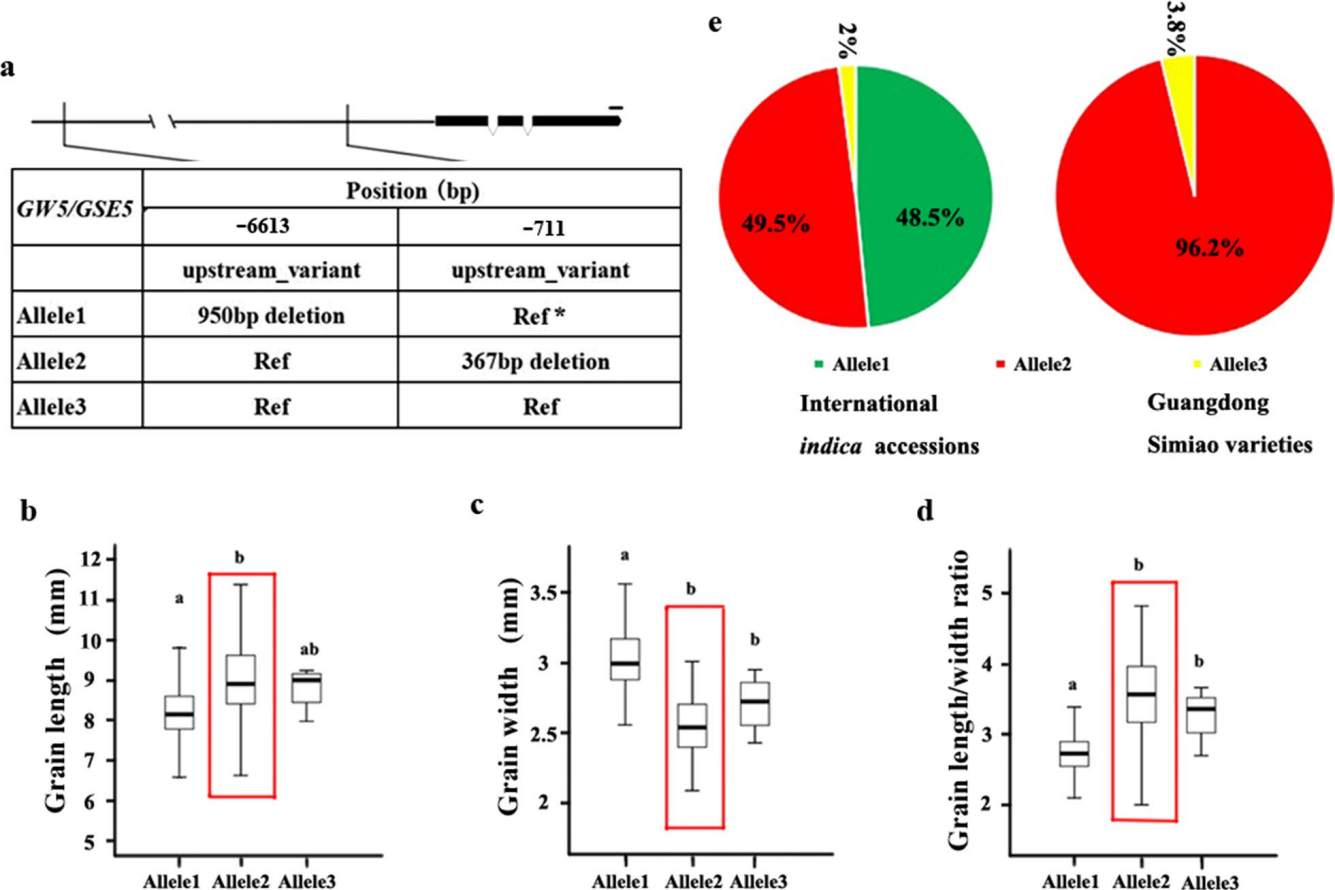
Allelic effects and distribution of *GW5/GSE5*. Alleles of *GW5/GSE5* (**a**); comparisons of grain length (**b**), grain width (**c**) and length/width ratio (**d**) for the three *GW5/GSE5* alleles; the distribution of *GW5/GSE5* alleles (**e**) in international *indica* accessions and Guangdong Simiao varieties; * A pangenome was used as the reference genome (Wang et al. 2022); letters above the bars are ranked by Duncan’s test at *P* < 0.05; different letters indicate significant differences; red rectangle box: major allele in Guangdong Simiao varieties; scale bar: 100 bp

**Fig. 6:**
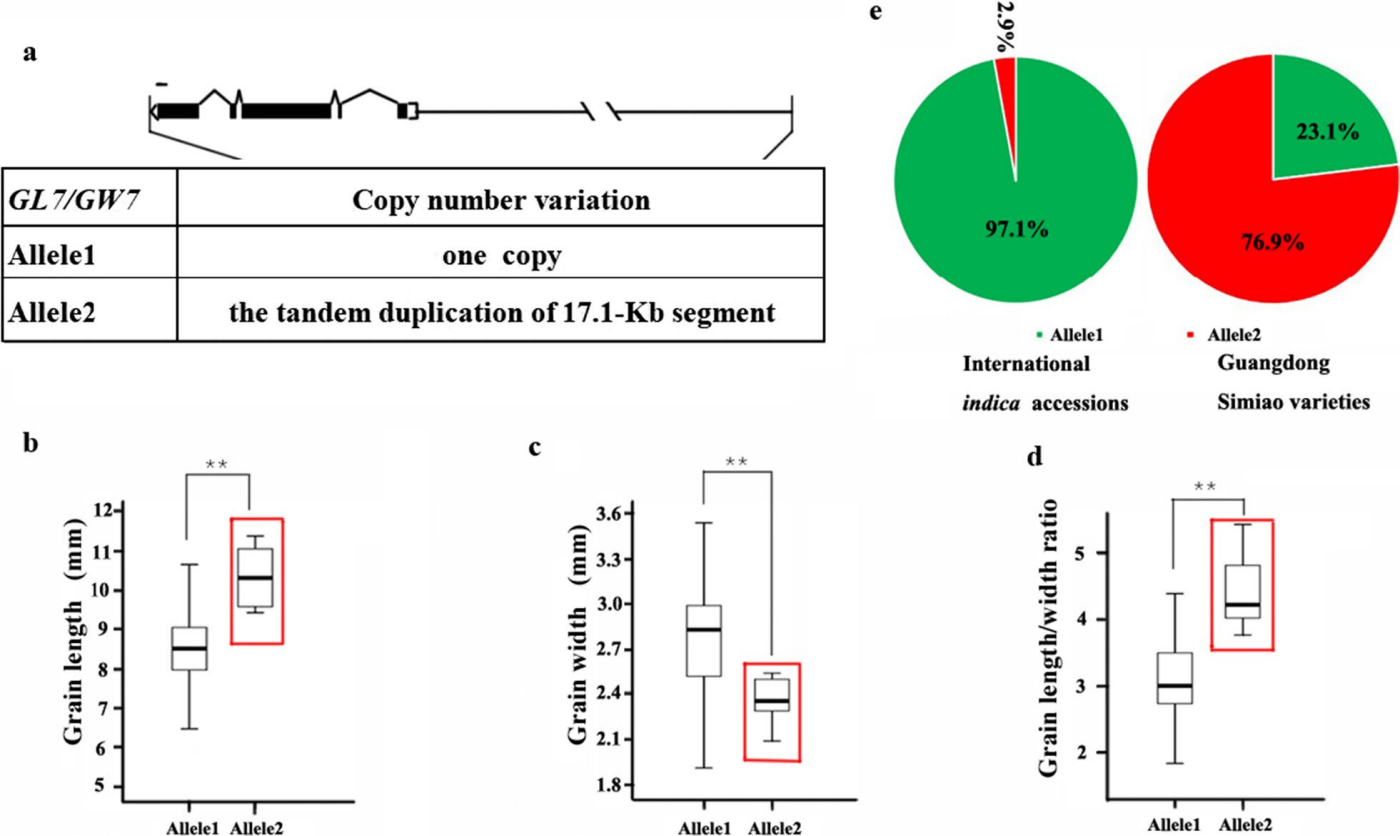
Allelic effects and distribution of *GL7/GW7*. Alleles of *GL7/GW7* (**a**); comparisons of grain length (**b**), grain width (**c**) and length/width ratio (**d**) for the two *GL7/GW7* alleles; the distribution of *GL7/GW7* alleles (**e**) in international *indica* accessions and Guangdong Simiao varieties; red rectangle box: major allele in Guangdong Simiao varieties; **, *P* < 0.01; scale bar: 100 bp

To further elucidate the selection patterns of grain shape genes, the alleles and distributions of the four selected genes (*GS3*, *GS5*, *GW5* and *GL7)* were further analyzed in both international *indica* accessions and Guangdong Simiao varieties based on their characterized functional variations (Figs. 3, 4, 5, 6).

For *GS3*, the SNPs that occurred in the second exon result in the early termination of the encoded protein and affect grain shape (Fan et al. 2006). In our results, two SNPs in the CDS region of *GS3* were found in the whole panel and three alleles could be identified (Fig. 3a). The accessions with allele 3 had longer and narrower grains and a higher length/width ratio than the accessions harboring allele 1 and allele 2 (Fig. 3b–d). Allele distribution analyses revealed that allele 3 accounted for 32.7% in international *indica* accessions and 96.2% in Guangdong Simiao varieties (Fig. 3e).

Sequence variations in the promoter region of *GS5* affect its expression, resulting in differences in grain length (Li et al. 2011). *GS5* had five alleles in the whole panel based on the seven variants present in the promoter region (Fig. 4a). The grain width and length/width ratio among the accessions carried different alleles of *GS5*, and the grain lengths of the accessions that carry allele 1, allele 3 and allele 4 were not significantly different, while the grain lengths between the accessions carried allele 2 and the accessions carried allele 5 were significantly different (Fig. 4b–d). The distribution of the five *GS5* alleles demonstrated that allele 2 accounted for 28.4% in international *indica* accessions and 74.5% in Guangdong Simiao varieties (Fig. 4e).

In *indica*, the presence or absence of a 950 bp sequence in the promoter region of *GW5/GSE5* was characterized as functional variation, which affected gene expression and led to different grain widths in rice (Duan et al. 2017; Liu et al. 2017). Using our developed pangenome pipeline (Wang et al. 2022), the presence/absence variations (PAVs) were characterized in the whole panel, and three alleles were identified (Fig. 5a). Accessions with allele 2 had longer and narrower grains and a higher length/width ratio than those accessions harboring allele 1 (Fig. 5b–d). Allele distribution analyses revealed that allele 2 accounted for 49.5% in international *indica* accessions and 96.2% in Guangdong Simiao varieties (Fig. 5e).

For *GL7*, the tandem duplication of the 17-kb interval was characterized as the causal variation conferring grain shape differences (Wang et al. 2015b). We determined the copy number of this 17-kb interval by mapping the resequencing data to the Nipponbare genome and then called the copy number using Mosdepth (Pedersen and Quinlan 2018). *GL7* was classified into two alleles based on the presence or absence of tandem duplication (Fig. 6a). Accessions with allele 2 (with the duplication) had longer and narrower grains and a higher length/width ratio than the accessions with allele 1 (without the duplication) (Fig. 6b–d). Allele distribution analyses revealed that allele 2 accounted for 2.9% in the international *indica* accessions and 76.9% in Guangdong Simiao varieties (Fig. 6e).

### The Allelic Combinations of the Four Genes and Their Grain Shape Phenotypes

To understand how the alleles of four major grain shape genes interact to forge the grain shape of Guangdong Simiao rice, the grain shape phenotypes of the rice accessions carrying different allelic combinations of the four selected genes were measured. In total, 30 allelic combinations were identified in the whole panel in the present study, with 28 allelic combinations found in the international *indica* accessions and 6 allelic combinations found in Guangdong Simiao varieties. The combinations found in more than 2 accessions were further analyzed in the present study (Additional files 3, 4: Tables S3, S4).

**Fig. 7:**
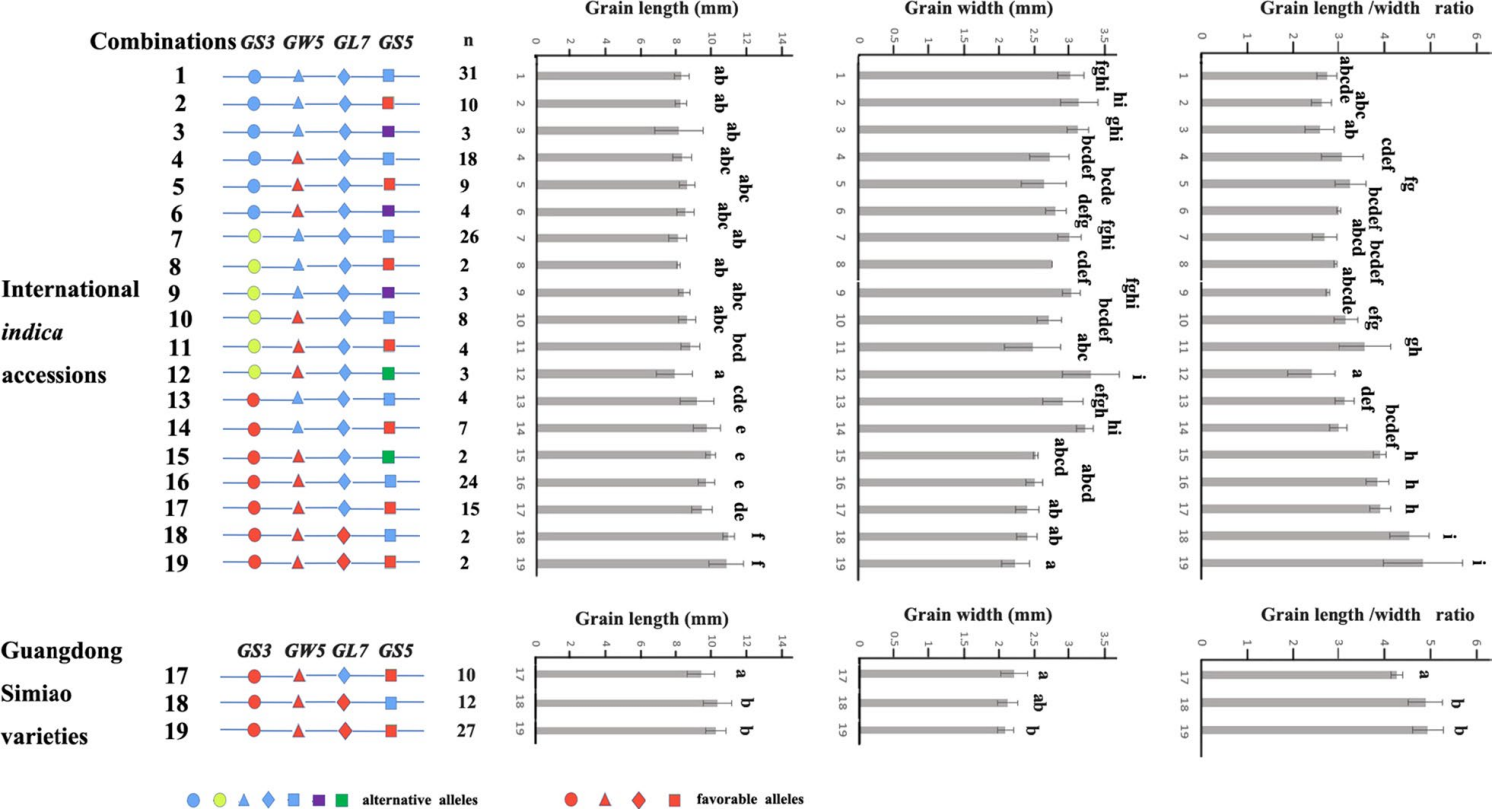
Major allelic combinations and phenotypes of the four grain shape genes. Alternative alleles: *GS3*^allele1^, *GW5*^allele1^, *GL7*^allele1^, *GS5*^allele1^, *GS3*^allele2^, *GS5*^allele3^, *GS5*^allele4^; according to the grain shape breeding goal of Simiao varieties, the alleles ( *GS3*^allele3^, *GW5*^allele2^, *GL7*^allele2^, *GS5*^allele2^) that produce long or/and slender grain shapes were defined as "favorable alleles". Letters above the bars are ranked by Duncan’s test at *P* < 0.05; different letters indicate significant differences; n, the number of strains (varieties)

In the international *indica* accessions, the accessions harboring combinations 18 (*GS3*^allele3^ + *GW5*^allele2^ + *GL7*^allele2^ + *GS5*^allele1^) and 19 (*GS3*^allele3^ + *GW5*^allele2^ + *GL7*^allele2^ + *GS5*^allele2^) had the longest grains (> 10.5 mm) and highest length/width ratio (> 4.5). The grain widths of the accessions harboring combinations 15, 16, 17, 18 and 19 were all below 2.6 mm (Fig. 7, Additional file 3: Table S3). In the Guangdong Simiao varieties, only three major allelic combinations were identified, namely, combinations 17, 18 and 19. Among them, combination 19 (*GS3*^allele3^ + *GW5*^allele2^ + *GL7*^allele2^ + *GS5*^allele2^) accounts for the highest proportion (51.9%), and the grain lengths of both combinations 18 and 19 exceed 10 mm with a length/width ratio exceeding 4.5. Combinations 17, 18 and 19 carried *GS3*^allele3^ and *GW5*^allele2^. Combinations 17 and 19 had different alleles at the *GL7* locus, and accessions with combination 17 (*GL7*^allele1^) had a significantly shorter grain length and lower length/width ratio than those with combination 19. Combinations 18 and 19 had different alleles at the *GS5* locus, but the differences in grain length, grain width and length/width ratio were not statistically significant (Fig. 7, Additional file 4: Table S4).

### Grain Shape Genes Selected for in the Guang 8B Pedigree

**Fig. 8:**
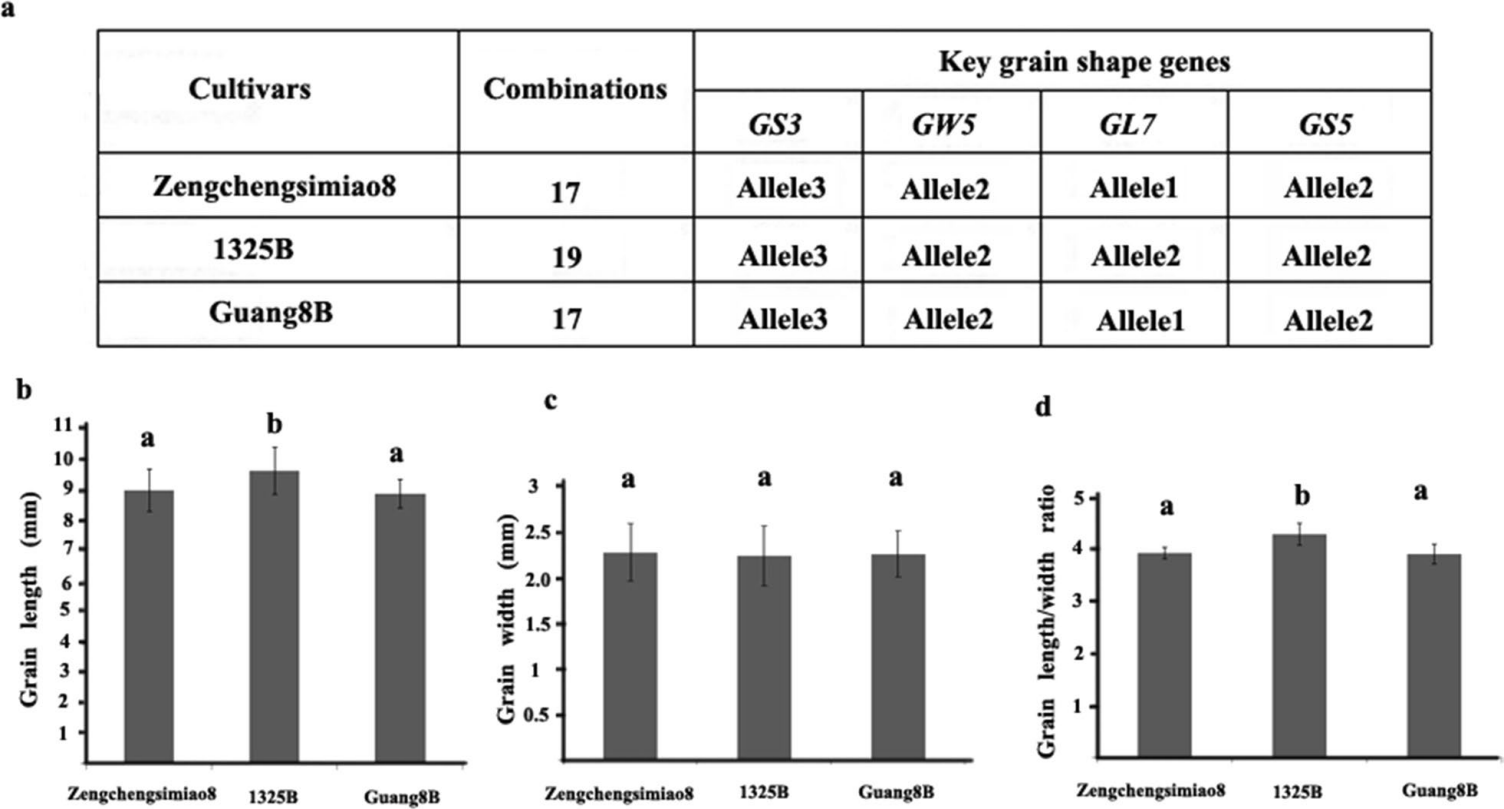
Grain shape gene combinations in the Guang8B pedigree. Allelic combinations of Guang8B and its parents (**a**); comparisons of grain length (**b**), grain width (**c**) and grain length/width ratio (**d**) for the three strains; letters above the bars are ranked by Duncan’s test at *P* < 0.05, different letters indicate significant difference

Guang 8A is the first Guangdong Simiao-type high-quality *indica* male sterile line released in China. Guang 8B is a maintainer line of Guang 8A. We analyzed the allelic combinations of the four grain shape genes described above in the Guang 8B pedigree to dissect how the selection process of these genes was involved in grain shape improvement during Guangdong Simiao variety breeding. In the Guang 8B pedigree, Zengchengsimiao 8 and 1325B are the direct parents of Guang 8B. Zengchengsimiao 8 is a landrace with small grains, which results in its high grain quality. Sequencing results demonstrated that all three lines carried the same allele combination of *GS3*, *GW5* and *GS5* (*GS3*^allele3^ + *GW5*^allele2^ + *GS5*^allele2^). However, the *GL7*^allele1^ allele derived from Zengchengsimiao 8 was selected during the breeding of Guang 8B, which led to its small grain phenotype. Due to the difference in *GL7* alleles, the parent Line 1325B (*GL7*^allele2^) had longer grains with a higher length/width ratio and a large grain shape, while the other parent Zengchengsimiao 8 and Guang 8B (*GL7*^allele1^) had significantly shorter grains with a reduced length/width ratio and a small grain shape (Fig. 8). The length/width ratios of the three rice lines exceeded 3.8 (brown rice length/width ratio exceeded 3.5), meeting the criteria of the Guangdong Simiao variety. Guang 8B represents a traditional Simiao variety with narrow and long grains. Despite its small grain, both Guang 8B and Zengchengsimiao 8 are also typical Simiao-type varieties, perhaps resulting from their *GS3*, *GW5* and *GS5* alleles.

## Discussion

### Key Grain Shape Genes were Selected During the Guangdong Simiao Rice Breeding Process

Rice grain shape varies according to local cuisine and culture; therefore, there are obvious regional characteristics for the grain shape preference of rice (Bai et al. 2010; Harberd 2015). In addition, grain shape is immediately obvious to consumers and is therefore a major factor in defining market value. Due to the importance of grain shape in rice production, much effort has been made in the genetic dissection of grain shape in rice in recent decades, and at least twenty genes conferring grain shape have been identified and functionally confirmed. However, current studies on the genetic dissection of grain shape have mainly focused on the function and effect of individual functional genes. Given that rice grain shape is a complex trait and is controlled by multiple genes, understanding the effects of different allele combinations of grain shape-regulating genes is of great practical significance to perform breeding by design of rice grain shape (Lee et al. 2015).

Simiao rice originated in Guangdong, China, is characterized by its slender grain, and is very popular in South China. To understand the genetics of grain shape of Guangdong Simiao varieties, we analyzed the selection signals of the twenty cloned genes conferring grain shape to identify the dominant genes contributed to Guangdong Simiao varieties and their alleles based on their sequence information in the present study. The results showed that the four genes (*GS3, GS5, GW5/GSE5* and *GL7/GW7*) fall into the selection region (Fig. 2a–d). Further allelic analysis revealed that the specific alleles of these genes (*GS3*^allele3^, *GW5*^allele2^, *GL7*^allele2^ and *GS5*^allele2^) account for much higher frequencies in the Guangdong Simiao than in diverse international *indica* accessions. All these alleles conferred longer or/and slender grain shapes in rice (Figs. 3, 4, 5, 6). These results suggest that these specific alleles of the four genes are strongly selected during the breeding process of Guangdong Simiao varieties. These results also implied that grain shape improvement may mainly be achieved by selecting the specific alleles of a handful of gain shape-related genes, which is meaningful for the future design of rice directional improvement.

### GS3 and GW5 are the Core Genes that Contribute to the Slender Grain Shape of the Guangdong Simiao Variety, and GL7 Determines the Grain Size

In previous studies, *GS3, GW5/GSE5* and *GL7/GW7* were all characterized by biparental population QTL mapping (Fan et al. 2006; Weng et al. 2008; Wang et al. 2015a, b) and have also been confirmed by GWAS analysis or allelic analysis. These results demonstrated that these genes might function across complex genetic backgrounds (McCouch et al.^2016^; Duan et al.^2017^; Wang et al.^2018^; Zhang et al.^2020^). In the present study, the allelic combination (*GS3*^allele3^ + *GW5*^allele2^) was as high as 96.2% in Guangdong Simiao varieties, indicating that these two genes play a core role in forging the slender grain shape of Guangdong Simiao varieties and have been intensively selected during the breeding process of Guangdong Simiao varieties. In addition to its crucial role in determining the grain shape of the Guangdong Simiao varieties, the effect of these two gene combinations (*GS3* + *GW5*) has also been confirmed to play an important role in the formation of rice grain shape in pyramiding lines with consistent genetic backgrounds or in natural populations with diverse genetic backgrounds. For instance, CSSLs with the target genes were developed by introgressing some preferable alleles (the alleles that produce the expected phenotypes) of *GS3* and *qSW5* derived from 9311 into the Teqing background. Three new elite cultivars with long and slender grains were obtained by gene pyramiding breeding using these CSSLs (Zeng et al.^2017^). By comparing the allelic combinations of *GS3* and *qSW5* harbored in 127 diverse rice germplasms, Lu et al. (2013) found that 39 varieties with a combination of *GS3*-A and *qSW5*-K produced long and slender grains, and 47 varieties that carried *GS3*-C and *qSW5*-N alleles had short and round grains. *GS3* was demonstrated to be the most important gene for grain length, whereas *qSW5* exerted the greatest effect on grain width, regardless of genetic background. It is rational that genes with large genetic effects, such as *GS3* and *qSW5,* can be easily selected and widely applied in breeding processes due to their large genetic effects (Lu et al. 2013; Zhang et al. 2021b). Our study provides evidence of this breeding selection from a population genetic point of view.

Our results further indicated that there were significant differences in grain shape between varieties with allele combinations 17 and 19 in Guangdong Simiao varieties. Combination 17 contains *GL7*^allele1^, while combination 19 contains *GL7*^allele2^. The grain length of the varieties with combination 17 was significantly shorter, and the length/width ratio was significantly lower than that of the varieties with combination 19. However, since they all contain the specific alleles of *GS3* and *GW5*, these varieties still met the criteria of the Guangdong Simiao variety in terms of grain length, grain width and length/width ratio. As a case study, we verified this pattern of grain shape gene selection in the Guang 8B pedigree, which led to the development of the Simiao-type sterile line Guang 8A. We found that both the grain length and the length/width ratio of Guang 8B were significantly reduced due to the inheritance of *GL7*^allele1^ from the parent Zengchengsimiao8 instead of *GL7*^allele2^ from the parent 1325B (Fig. 8). Therefore, allelic variation in *GL7* may be selected as the key factor in determining the grain size of Simiao varieties.

Although there were differences in alleles of *GS5* between combinations 18 (*GS5*^allele1^) and 19 (*GS5*^allele2^), the differences in grain shape between accessions with the two combinations were not significant (Fig. 7). However, it seems that breeders preferred selecting *GS5*^allele2^ (74.5%) in Guangdong Simiao varieties. The mechanism underlying this may be that *GS5* and *GW5* are closely linked (approximately 2 Mb long in distance), and alleles can easily be simultaneously selected in breeding. However, we cannot rule out the possibility that *GS5*^allele2^ could be responsible for other desired traits in Guangdong Simiao varieties. More studies are needed to clarify these issues.

In general, the final size of rice grains is coordinately controlled by cell proliferation and cell expansion. In addition to their large genetic effect, the artificial selection of the effective allelic combinations during grain shape improvement of Guangdong Simiao varieties may also imply that the selected alleles of the four grain shape genes may play nonredundant roles and mechanisms in grain shape regulation. *GS3* is a G protein γ-subunit (G_γ_) that is functionally differentiated due to a variation in the C-terminal structural domain of its protein; it binds G_β_ competitively with DEP1 or GGC2, resulting in shorter grain length (Sun et al. 2018). *GW5* protein can physically interact with and repress the kinase activity of rice GSK2, resulting in the accumulation of unphosphorylated OsBZR1 and DLT proteins in the nucleus to mediate brassinosteroid (BR)-responsive gene expression and growth responses (Liu et al. 2017). A study found that *GS5* regulates grain size by preventing OsBAK1-7 endocytosis and enhancing BR signaling, suggesting a possible link between *GS5* and BR signaling in grain size control (Xu et al. 2015). *GL7/GW7* encodes a TON1 RECRUITING MOTIF (TRM)-containing protein homologous to Arabidopsis LONGIFOLIA proteins involved in microtubule regulation. It was shown that *OsSPL16/GW8* binds to the promoter of *GL7/GW7* and represses its transcription to regulate cell proliferation in the spikelet hull (Wang et al. 2015a). Therefore, *GL7* may be a transcriptional regulation factor (Li et al. 2018). These results suggest that *GS3, GS5, GW5* and *GL7* control grain size through independent signal regulation pathways. This may be one of the reasons why these four genes were selected together to confer complete regulation of grain shape.

### Directional Improvement of Grain Shape of Guangdong Simiao Varieties

Guangdong Simiao varieties not only require long and slender grain shapes but also have an excellent appearance quality. Low chalkiness is another important indicator of Guangdong Simiao varieties (T/GDSMM 002-2019). It has been reported that the transparent grain (low chalkiness) of Guangdong Simiao varieties is partially attributed to their slender grains (Zhou et al. 2022). However, varieties with small grains tend to have a lower thousand grain weight and yield (Lu et al. 2013). High yield and good quality are long-standing contradictions for rice breeding because improvements in one are often associated with detrimental effects on the other (Harberd 2015). Benefiting from the cloning and mechanistic dissection of a few favorable genes, this contradiction has been largely alleviated. According to our results, 75% of the gene combinations (*GS3*^allele3^ + *GW5*^allele2^ + *GL7*^allele2^) in Guangdong Simiao varieties had a large grain phenotype, and 19.2% of the gene combinations (*GS3*^allele3^ + *GW5*^allele2^ + *GL7*^allele1^) had a small grain phenotype. The varieties carrying *GL7*^allele2^ appeared to have larger grains, as well as larger and denser starch granules, which significantly helped reduce the chalkiness of rice (Wang et al. 2015a ,b), and the varieties with large grains tended to be more advantageous in yield; therefore, *GL7* can coordinate yield and quality well and is an important gene in Guangdong Simiao varieties. Thus, the frequently observed negative correlation can be broken by the use of some specific allele of functional genes (Harberd 2015). Therefore, combination 19 (*GS3*^allele3^ + *GW5*^allele2^ + *GL7*^allele2^ + *GS5*^allele2^) was the optimal choice in terms of balancing yield and quality traits in breeding for Guangdong Simiao varieties. Most of the Guangdong Simiao varieties released in recent years, such as 19 Xiang and Xiangzhuxiangsimiao, harbored this allelic combination, and they are all elite parents for developing new Simiao varieties.

## Conclusion

In the present study, a comprehensive investigation of population-wide selection pattern and allele combination of grain shape genes was conducted to dissect the molecular genetic mechanisms underlying the grain shape improvement of Guangdong Simiao varieties. Our results revealed that *GS3*, *GS5*, *GW5* and *GL7* are the key genes selected in Guangdong Simiao varieties for grain shape improvement. We also demonstrated that combination 19 (*GS3*^allele3^ + *GW5*^allele2^ + *GL7*^allele2^ + *GS5*^allele2^) was the predominant allelic combination in Guangdong Simiao varieties. Since the present Guangdong Simiao varieties have great potential to be used as elite parents for the subsequent development of new superior high-quality rice varieties, dissecting the genetic basis of grain shape improvement in the present study is of great significance for future molecular breeding. The present study also provided valuable genomic and genetic insights into grain shape improvement and the coordination of yield and quality in rice by molecular breeding. Furthermore, these results shed light on future investigations of rice breeding processes using allelic and genomic information, as well as guide directional breeding scheme design.

## Materials and Methods

### Plant Materials

A total of two hundred nineteen international *indica* accessions were selected from RDP2 according to their diversity and representation (McCouch et al. 2016). Fifty-two Guangdong Simiao varieties and two parents of Guang8B were collected and selected from the Rice Research Institute of Guangdong Academy of Agricultural Sciences. These test materials were planted by strain at the Dafeng research base of the Rice Research Institute of Guangdong Academy of Agricultural Sciences (Additional file 1: Table S1). The seeds were harvested by strain 35 days after heading and dried for phenotype determination. There were three replicates for each rice strain.

### Determination of Grain Size

The grain length, grain width and length/width ratio of each strain (variety) were determined by using a Wanshen automatic grain analyzer (Hangzhou Wanshen Testing Technology Co., Ltd., Hangzhou, Zhejiang, China), taking 100 full seeds of each strain, scanning and reading the three indices of grain length, grain width and grain length/width ratio, and repeating the determination three times for each strain.

### Genome Sequencing and Fixation Index Analysis

The leaves of rice seedlings were collected and subjected to DNA extraction by the CTAB method. Sequencing was performed on the Illumina NovaSeq6000 platform. A fastx_toolkit (http://hannonlab.cshl.edu/fastx_toolkit) was used to remove adaptor and low-quality reads. All reads have been deposited in the NCBI sequence read archive (BioProject accession PRJNA820969). Short read resequencing data were aligned to the Nipponbare reference genome using BWA-MEM (Li and Durbin 2009). The results were sorted using Picard and filtered using SAMtools (Li et al. 2009), retaining reads with a mapping quality over 20. Nucleotide variants for each accession were detected using HaplotypeCaller in GATK (v3.8-1-0) with the default parameters. Population nucleotide variants were called using the Combine GVCFs and Genotype GVCFs tool in GATK (McKenna et al.^2010^). All genotypes were filtered using the Select Variants and Variant Filtration tool in GATK. Fixation index (Fst) analysis between international *indica* accessions and Guangdong Simiao varieties was conducted using a 100-kb sliding window (with a 10-kb step for Fst value calculation) using VCF tools (Danecek et al. 2011).

### Allele Mining

Alleles of *GS3*, *GW5*, *GS5* and *GL7* of all accessions of the population were extracted for genotypes called by resequencing data from the VCF file obtained above. Using our developed pangenome pipeline PSVCP (Wang et al. 2022), the presence/absence variations (PAVs) of the *GW5* locus were characterized in the whole panel. Since the functional allele of *GL7* is correlated with its copy number, we assessed the copy number of *GL7* by using short-read sequencing data and Mosdepth (Pedersen and Quinlan 2018).

### Data Analysis

Significance testing of differences between 2 groups of data was performed using the *t test* module of *SPSS*; significance testing of differences among 3 and more groups of data was performed using the Duncan’s multiple range comparison module of *SPSS*; and multiple linear regression analysis were performed using the *SPSS* to assess the contribution of genes to phenotype variation.

## Supplementary Information

**Additional file 1: Table S1.** Grain shape and information of 219 international *indica* accessions and 52 Guangdong Simiao varieties.

**Additional file 2: Table S2.** The fixation index of genomic regions harboring sixteen rice grain shape genes.

**Additional file 3: Table S3.** The allelic combinations of international *indica* accessions.

**Additional file 4: Table S4.** The allelic combinations of Guangdong Simiao varieties.

## Data Availability

The datasets supporting the conclusions of this article are provided within the article and its additional files. The raw read data (FASTQ files) of all accessions used in the present study were uploaded to NCBI’s sequence read archive (BioProject accession PRJNA820969).

## Abbreviations

GWAS: Genome wide association analysis
CSSL: Chromosome segment substitution line
RDP2: Rice diversity germplasm platform2
CDS: Coding sequence
SNP: Single nucleotide polymorphism
QTL: Quantitative trait loci
PAV: The presence/absence variation
Fst: Fixation index
DNA: Deoxyribonucleic acid
NCBI: US national center for biotechnology information
